# Prevalence, risk factors, and optimized management of moderate-to-severe thirst in the post-anesthesia care unit

**DOI:** 10.1038/s41598-020-73235-5

**Published:** 2020-09-30

**Authors:** Chia-Wei Lee, Shih-Ting Liu, Ya-Jung Cheng, Ching-Tang Chiu, Yu-Fen Hsu, Anne Chao

**Affiliations:** 1 Department of Anesthesiology, National Taiwan University Hospital, Taipei, 10002 Taiwan; 2 Department of Nursing, National Taiwan University Cancer Center, Taipei, 10672 Taiwan

**Keywords:** Health care, Medical research, Risk factors

## Abstract

Post-operative thirst is common and may cause intense patient discomfort. The aims of this retrospective study conducted in a high-volume post-anesthesia care unit (PACU) were as follows: (1) to examine the prevalence of moderate-to-severe post-operative thirst—defined as a numerical rating scale (NRS) score of 4 or higher, (2) to identify the main risk factors for moderate-to-severe post-operative thirst, and (3) to maximize the efficacy and safety of thirst management through a quality improvement program. During a 1-month quality improvement program conducted in August 2018, a total of 1211 adult patients admitted to our PACU were examined. Moderate-to-severe thirst was identified in 675 cases (55.8%). The use of glycopyrrolate during anesthesia was associated with moderate-to-severe thirst (71.7% versus 66.4%, respectively, p = 0.047; adjusted odds ratio: 1.46, p = 0.013). Following a safety assessment, ice cubes, room temperature water, or an oral moisturizer were offered to patients. A generalized estimating equation model revealed that ice cubes were the most effective means for thirst management—resulting in an estimated thirst intensity reduction of 0.93 NRS points at each 15-min interval assessment (p < 0.001)—followed by room temperature water (− 0.92/time-point, p < 0.001) and the oral moisturizer (− 0.60/time-point; p < 0.001). Patient satisfaction (rated from 1 [definitely dissatisfied] to 5 [very satisfied]) followed a similar pattern (ice cubes: 4.22 ± 0.58; room temperature water: 4.08 ± 0.55; oral moisturizer: 3.90 ± 0.55, p < 0.001). The use of glycopyrrolate—an anticholinergic agent that reduces salivary secretion—was the main independent risk factor for moderate-to-severe post-operative thirst. Our findings may provide clues towards an optimized management of thirst in the immediate post-operative period.

## Introduction

Post-operative thirst is common (reported incidence of the moderate-to-severe form: 53.2–69.8%)^1,2^ and may cause intense patient discomfort. Unfortunately, its clinical relevance is frequently downplayed, being generally considered less distressing than other post-operative symptoms such as pain, nausea, vomiting, or sore throat.

The etiology of post-operative thirst is multifaceted and has not been yet completely elucidated. Among various pathophysiologic factors, preoperative fasting and perioperative fluid loss may lead to hyperosmolarity and hypovolemia. Moreover, certain drugs utilized in anesthetic practice (e.g., glycopyrrolate and other anticholinergic agents used for reducing salivary secretion) may promote a thirst sensation, which may be further intensified by prolonged surgical and intubation times^3^. Several possible approaches have been proposed to quench thirst—including low temperature, menthol in association with cold strategies, chewing gums, acupressure, early fluid ingestion, substitute saliva, and the use of a thin straw^4^. The choice among such strategies is at least in part dependent on the clinical setting. For example, low temperature is deemed suitable in the post-operative care unit (PACU)—serving the dual purpose of limiting the ingested volume while stimulating cold-sensitive receptors^5,6^. There is growing clinical interest in improving the ability to quench post-operative thirst and in optimizing thirst management.

In our high-volume PACU, thirst has been traditionally managed using wet cotton swabs. However, the increasing number of complaints for moderate-to-severe post-operative thirst prompted us to improve our traditional management strategies through an ad hoc quality survey. In this scenario, we designed the current study with the following three aims: (1) to examine the prevalence of moderate-to-severe post-operative thirst—defined as a numerical rating scale (NRS) score of 4 or higher, (2) to identify the main risk factors for moderate-to-severe post-operative thirst, and (3) to maximize the efficacy and safety of thirst management through a quality improvement program.

## Methods

### Ethical approval

This retrospective study was approved by the Research Ethics Committee of the National Taiwan University Hospital, which waived the need for written consent (No 201809050RINC). All procedures were in accordance with current guidelines and regulations.

### Development and testing of a thirst management quality improvement program

A literature review was performed to identify feasible thirst-relieving strategies and safety protocols. Two anesthesiologists and the head nurse in charge of the PACU designed the thirst record chart and the management model. They also convened PACU meetings to discuss thirst assessment, the safety protocol for thirst management, and different thirst interventions before initiating the program. After meetings and appropriate environmental scans, thirst treatment with water, ice cubes, and oral moisturizers (wet swabs or gargling) were considered suitable for our PACU setting. A 10-day pilot testing was started before implementing the program with the following goals: (1) to observe patient response to thirst management; (2) to ensure that all of the nurses in PACU were capable of assessing thirst intensity using the NRS score and performing safety assessment; (3) to estimate the personnel required to offer the thirst management program in a timely manner; and (4) to provide adequate feedback to the nurses in an effort to optimize the program.

### Study setting and participants

The study data were retrospectively collected over a 1-month quality improvement program implemented during August 2018. The study was conducted in the National Taiwan University Hospital, a public tertiary medical center located in Taipei, Taiwan. The principal operating theater consists of 25 operating rooms, with an adjacent 12-bed PACU. Upon completion of surgery, patients are routinely transferred to the PACU where they are monitored for at least one hour. The number of annual PACU admissions in our hospital is approximately 20,000. The nurse-to-patient ratio varies between 1:2 to 1:4, depending on work shifts and patient clinical conditions. Patients aged at least 20 years were included and classified according to surgery type in one of the following categories: general surgery, otolaryngology, thoracic surgery, urology, neurology, plastic surgery, and orthopedic surgery. Based on the nursing system implemented in our PACU, patients who underwent dental, eye, and peripheral vascular surgical procedures were grouped together into a unique “other surgery” category. Patients who underwent pediatric, obstetrics and gynecology surgery were followed in National Taiwan University Children’s Hospital and were not considered for inclusion. We also excluded subjects who were unable to communicate or understand instructions clearly and those whose oral intake required strict restriction during the post-surgical phase. Patient demographic and clinical characteristics—including age, sex, anesthesia technique and medications—were extracted from digital clinical records.

### Thirst management

Different options for thirst management were available − including ice cubes (one ice cube obtained from 10 mL distilled water), room temperature distilled water (10 mL), or an oral moisturizer (gargling or wet swab). Ice cubes and water were prepared in small cups. Patients assigned to the oral moisturizer were asked to hold in their mouth for 10–15 s either room temperature distilled water (10 mL) or a large cotton swab (cotton ball diameter: ~ 12 mm) previously soaked in distilled water.

In the context of the quality survey, patients were offered with only one option selected by nurses in charge. In general, we alternatively offered an ice cube or room temperature water to each patient with thirst who passed the safety assessment. Oral moisturizer was given to (1) thirsty patients who refused an ice cube or water but nonetheless required some moisture or (2) patients who were not allowed to swallow water but were deemed eligible for an oral moisturizer in the PACU. For safety reasons, water and ice cubes were provided with an equal volume limit (10 mL) at each time point (0, 15, 30, and 45 min). No other oral intake was permitted within each 15-min interval. Consequently, the maximum oral fluid intake allowed in our PACU did not exceed 40 mL.

### Measurements used for the quality improvement program

The following measures were implemented upon PACU admission (0 min) and at 15, 30, and 45 min post-admission: assessment of thirst intensity and application of a safety protocol for thirst management. The intensity of thirst was graded on a numerical rating scale (NRS) ranging from 0 (no thirst) to 10 (worst thirst). Patients were asked to respond to the following question: “If 0 is no thirst and 10 is the worst thirst you can imagine; how would you rate your thirst right now?” This approach has been extensively applied to assess subjective pain intensity and found to be valid^7^. Moreover, it has been previously used to investigate thirst intensity in the intensive care unit and PACU settings^8,9^. Notably, the reliability of this method was confirmed in our study (Cronbach’s α = 0.822 for thirst scores recorded at the four time points).

Before implementing measures for thirst relief, a modified Safety Protocol for Thirst Management (SPTM)^10^ consisting of standardized questions was applied to all participants. The protocol consisted of the following three safety criteria: (1) consciousness, (2) airway reflex, and (3) sensation of nausea. In presence of at least one failed item, the patient was not allowed to receive thirst management for 15 min until the subsequent assessment. Patients who passed SPTM assessments were allowed to accept or decline the thirst management at each time point. In case of sleepiness or reduced level of consciousness, the nurse in charge stopped the management and reassessed the patient 15 min thereafter.

Before discharge from PACU, patient satisfaction with the thirst management intervention was rated on a 5-point Likert scale, as follows: 1, definitely dissatisfied; 2, somewhat dissatisfied; 3, neutral; 4, satisfied; and 5, very satisfied. Likert scales have been extensively utilized to assess post-operative satisfaction in various domains—including fluid management^11,12^. In addition, they are rapid, easy-to-use tools that do not significantly increase nursing workload in a high-volume PACU.

### Statistical analysis

Analyses were conducted on a dataset of 1211 adult patients. Although no accepted threshold still exists for thirst management, we selected a NRS ≥ 4 cutoff in accordance with a previous study showing that a value of 4 may identify patients with clinically relevant burden and also distinguish between mild and moderate thirst^13^. Scores were subsequently categorized as follows: 0, no thirst; 1–3, mild thirst; 4–6, moderate thirst; 7–10, severe thirst. For the purpose of analysis, patients were divided into two groups based on their thirst intensity before any intervention: none-to-mild thirst (NRS scores from 0 to 3) and moderate-to-severe thirst (NRS scores from 4 to 10).

Comparisons of continuous variables in patients with none-to-mild thirst versus moderate-to-severe thirst were performed using the Student’s *t*-test. Three-group comparisons of continuous data were conducted using one-way analysis of variance (ANOVA). Categorical variables were analyzed with the chi-square test or the Fisher’s exact test, as appropriate. Variables with a P value < 0.2 in univariate analysis were included as covariates in multivariable analysis^14^. Multivariable binary logistic regression analysis was conducted to identify the independent predictors of moderate-to-severe thirst at the first assessment. While the robustness of the logistic regression model might be suboptimal with small sample size, the sample size of our study was sufficiently large to obtain reliable results^15^. “Regional anesthesia” and “anesthesia without glycopyrrolate use” served as reference categories. The logistic regression model was well-fitting (p > 0.05; Hosmer–Lemeshow goodness-of-fit test). The performance of the model was assessed using receiver operating characteristic (ROC) curve analysis. Results are expressed as odds ratios (ORs) with their 95% confidence interval (CIs).

Among patients who received thirst management, changes in thirst intensity were estimated using a generalized estimating equation (GEE) model after adjustment for age, sex, surgery type, anesthesia type, nothing-by-mouth duration, American Society of Anesthesiologists classification, operating time, body temperature, perioperative fluid balance, and perioperative medication use (atropine, glycopyrrolate, ketamine, neostigmine, and furosemide) with a first order autoregressive [AR (1)] correlation structure. Specifically, the AR (1) correlation structure assumes a higher degree of correlation for measurements taken closer in time. Both patients with none-to-mild and those with moderate-to-severe thirst were included in the analysis. The GEE method is used to estimate parameters of a generalized linear model when a possible unknown correlation between outcomes exists^16,17^. Because each patient in our study had four thirst intensity scores, the degree of data collinearity was high. In keeping with the methodology of longitudinal studies, we considered different time points at which assessments were made as a continuous variable to model the interaction between time and thirst management. Moreover, our retrospective study had some missing thirst intensity scores (in patients with sleepiness or reduced level of consciousness). In this scenario, GEE offers a viable option for missing data handling in that it provides more accurate statistical inferences compared with repeated-measures ANOVA^18^. Two-tailed p values < 0.05 were considered statistically significant. Statistical analyses were conducted in IBM SPSS Statistics 23 (IBM Corp, Armonk, NY, USA).

## Results

**Figure 1:**
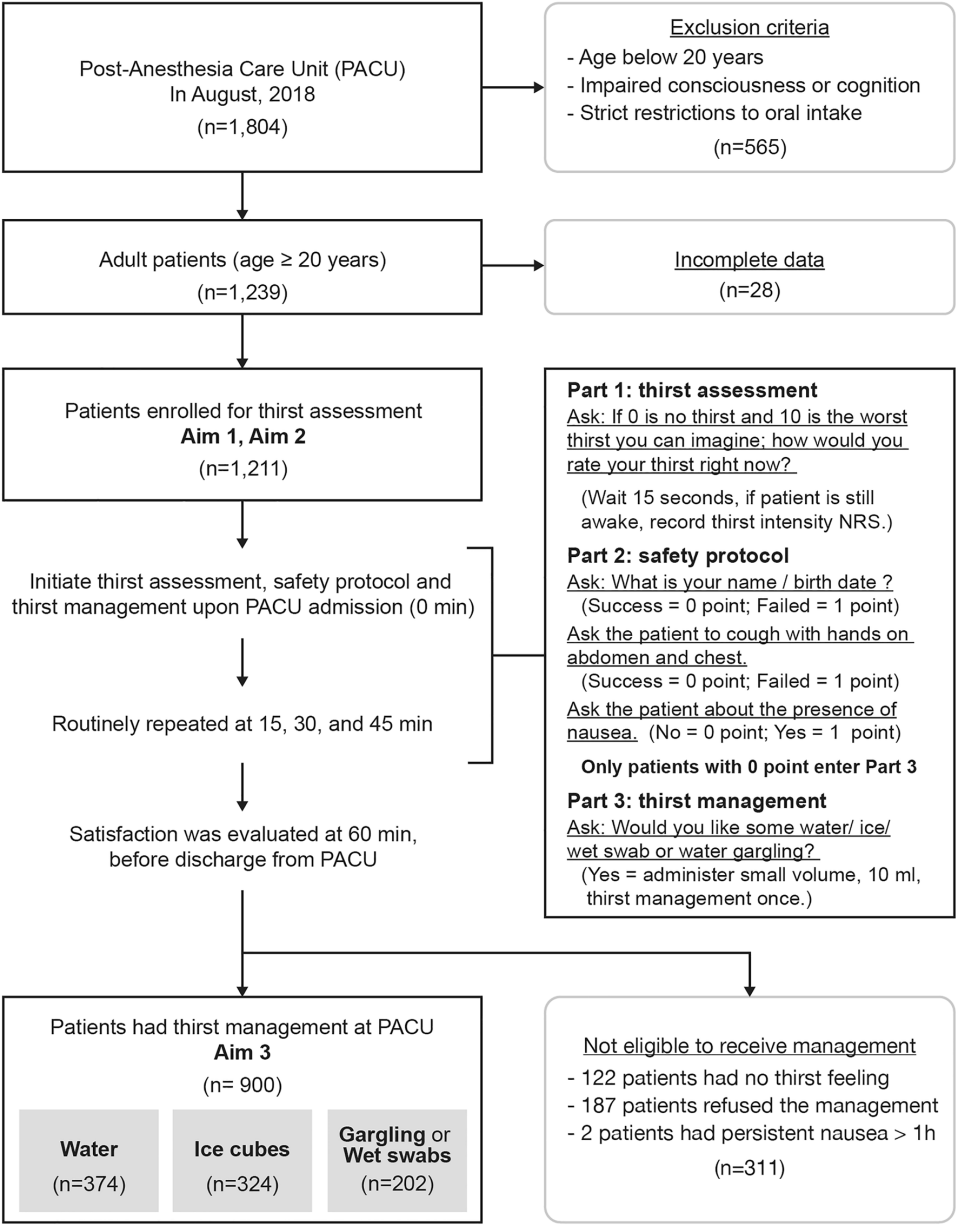
Flow of patients through the study.

Figure 1 depicts the flow of patients through the study. Of the 1804 patients who presented in our PACU during August 2018, 565 met the following exclusion criteria: (1) age below 20 years, (2) impaired consciousness or cognition, or (3) strict restrictions to oral intake. Of the remaining 1239 patients, 28 were further excluded because of incomplete data. Therefore, the sample for the first two study aims consisted of 1211 patients. Of them, 311 were excluded from the thirst management study for the following reasons: no thirst feeling (n = 122); treatment refusal despite thirst feeling (n = 187, of whom 153 patients had mild and 34 moderate-to-severe thirst); and failure of safety protocol for persisting nausea (n = 2). Therefore, the third study aim was assessed in 900 patients who received thirst management in the PACU.

### Prevalence of post-operative thirst

The prevalence of post-operative thirst during the first PACU assessment was as follows: no thirst, n = 247 (20.4%); mild thirst, n = 289 (23.9%); moderate thirst, n = 439 (36.3%); and severe thirst, n = 236 (19.5%). Only 123 patients (10.2%) consistently maintained a “no thirst” status during their four PACU assessments.

### Factors associated with moderate-to-severe post-operative thirst

Moderate-to-severe thirst (NRS scores from 4 to 10) during the first PACU assessment was present in 675 (55.8%) patients. Table 1 depicts the general characteristics of subjects with none-to-mild thirst versus moderate-to-severe thirst. The use of glycopyrrolate during anesthesia was significantly more frequent in patients who experienced moderate-to-severe thirst than in those with none-to-mild thirst (71.7% versus 66.4%, respectively, p = 0.047). In univariate analysis, the following variables showed a p < 0.2 for an association with moderate-to-severe thirst and were included as covariates in multivariable analysis: anesthesia type, use of glycopyrrolate, operating time, and peri-operative fluid balance.

**Table 1:** General characteristics of patients (n = 1211) with moderate-to-severe (NRS scores from 4 to 10) versus none-to-mild (NRS scores from 0 to 3) post-operative thirst at first assessment.

Variable	Moderate-to-severe thirst (n = 675)	None-to-mild thirst (n = 536)	p-value
**Demographic variables**			
Age, years	56.81 ± 15.74	57.16 ± 16.16	0.702
Sex, n (%)			0.721
Male	357 (52.89%)	289 (53.92%)	
Female	318 (47.11%)	247 (46.08%)	
ASA classification, n (%)			0.345
I	49 (7.26%)	35 (6.53%)	
II	404 (59.85%)	300 (55.97%)	
III	218 (32.30%)	199 (37.13%)	
IV	4 (0.59%)	2 (0.37%)	
Emergency surgery, n (%)			0.597
No	639 (94.67%)	511 (95.34%)	
Yes	36 (5.33%)	25 (4.66%)	
Time of last food intake, h	14.34 ± 3.76	14.37 ± 5.20	0.924
**Operation-related variables**			
Surgery type, n (%)			0.288
Urological surgery	150 (22.22%)	129 (24.07%)	
Orthopedic surgery	134 (19.85%)	94 (17.54%)	
General surgery	108 (16.00%)	83 (15.49%)	
Otolaryngology surgery	98 (14.52%)	83 (15.49%)	
Thoracic surgery	75 (11.11%)	66 (12.31%)	
Plastic surgery	40 (5.93%)	36 (6.72%)	
Neurosurgery	38 (5.63%)	34 (6.34%)	
Other types of surgery	32 (4.74%)	11 (2.05%)	
Types of anesthesia, n (%)			0.031
General (with ETT)	387 (57.33%)	326 (60.82%)	
General (IVGA)	134 (19.85%)	108 (20.15%)	
General (with SGA)	81 (12.00%)	43 (8.02%)	
Regional	73 (10.81%)	59 (11.01%)	
Atropine use, n (%)	96 (14.22%)	74 (13.81%)	0.836
Glycopyrrolate use, n (%)	484 (71.70%)	356 (66.42%)	0.047
Neostigmine use, n (%)	364 (53.93%)	295 (55.04%)	0.700
Ketamine use, n (%)	56 (8.30%)	53 (9.89%)	0.394
Furosemide use, n (%)	11 (1.63%)	9 (1.68%)	0.947
Operating time, min	82.58 ± 71.24	88.65 ± 89.16	0.200
Peri-operative fluid balance, ml	475.96 ± 422.29	520.63 ± 477.96	0.089
Body temperature, n (%)			0.340
Normal	583 (86.37%)	477 (88.99%)	
Hyperthermal	80 (11.85%)	53 (9.89%)	
Hypothermal	12 (1.78%)	6 (1.03%)	

ASA, American Society of Anesthesiologists; ETT, endotracheal tube; SGA, supraglottic airway device; IVGA, intravenous general anesthesia.

p values were calculated by independent Student’s *t*-tests or chi-square tests, as appropriate.

Notably, the use of glycopyrrolate was the only independent predictor of moderate-to-severe thirst identified on multivariable binary logistic regression analysis (adjusted odds ratio: 1.46, p = 0.013; Table 2). The Hosmer–Lemeshow goodness-of-fit test for logistic regression was not significant (p = 0.844). Moreover, the area under the ROC curve was 0.864, indicating good discriminative ability.

**Table 2:** Multivariable binary logistic regression analysis for independent predictors of moderate-to-severe post-operative thirst at the first assessment.

**Variable**	Odds ratio	95% confidence interval		P value
		Lower bound	Upper bound	
**Anesthesia type**				
General (with ETT)	0.73	0.47	1.14	0.169
General (IVGA )	0.81	0.51	1.27	0.356
General (with SGA)	1.12	0.64	1.94	0.695
Regional	1 (ref)			
**Use of glycopyrrolate**				
Yes	1.46	1.08	1.98	0.013
No	1 (ref)			
**Operating time**	1.00	1.00	1.00	0.828
**Peri-operative fluid balance**	1.00	1.00	1.00	0.293

ETT, endotracheal tube; SGA, supraglottic airway device; IVGA, intravenous general anesthesia; ref, reference group.

### Thirst management optimization program

**Figure 2:**
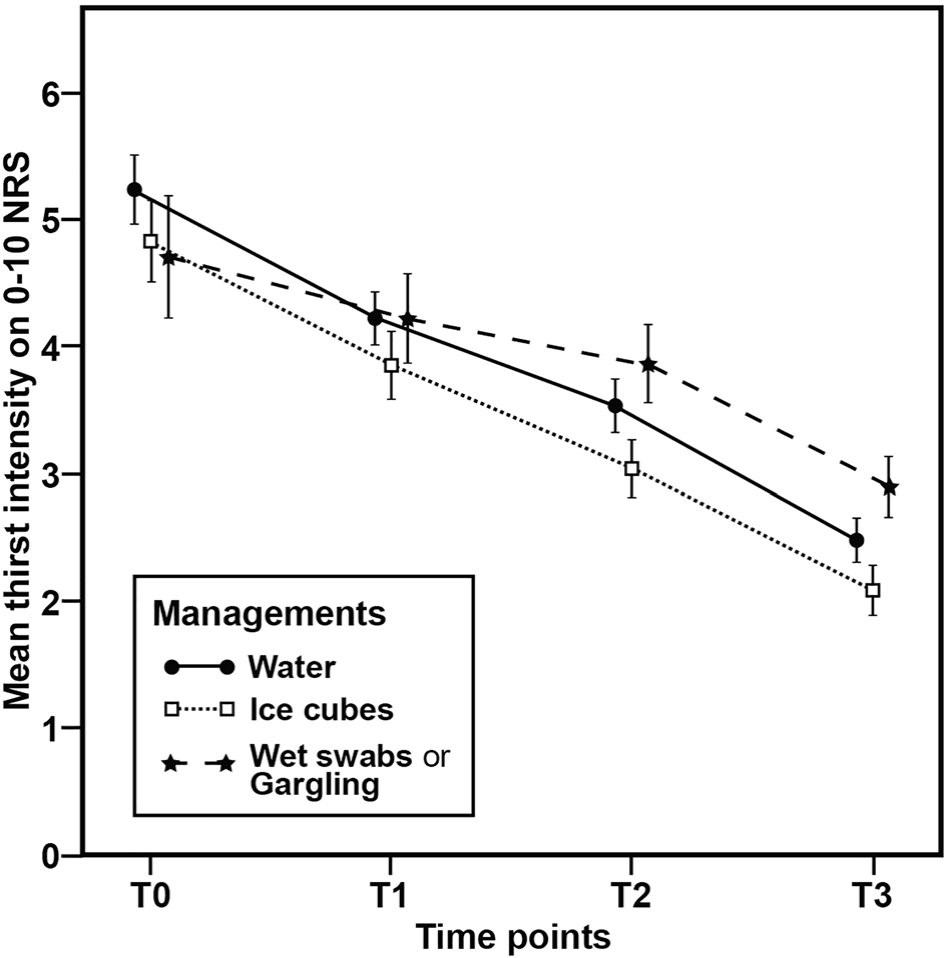
Intensity of post-operative thirst assessed at four different time points (i.e., every 15 min for 1 h) in the three management strategy groups: room temperature water (n = 376), ice cubes (n = 324), and oral moisturizers (n = 202).

A total of 900 patients entered into the thirst management optimization program. The prevalence of patients who fulfilled safety criteria for thirst management ranged between 78.22% and 99.50%, and the pattern was characterized by an increase over time (Table 3). Conversely, there was a temporal decrease in the number of patients who received thirst management. Table 4 shows the estimated changes in thirst intensity according to the GEE model. As expected, all of the three management approaches (i.e., ice cubes, room temperature water, oral moisturizer) significantly decreased NRS scores over subsequent assessments in PACU. Notably, ice cubes were the most effective means for thirst management—resulting in an estimated thirst intensity reduction of 0.93 NRS points at each 15-min interval assessment (p < 0.001)—followed by room temperature water (− 0.92/time-point, p < 0.001), and the oral moisturizer (− 0.60/time-point; p < 0.001). Patient satisfaction followed a similar pattern (ice cubes: 4.22 ± 0.58; room temperature water: 4.08 ± 0.55; oral moisturizer: 3.90 ± 0.55, p < 0.001). Figure 2 and Table 5 show that NRS scores were consistently reduced to values lower than 3. Compared with initially observed scores (NRS ≥ 4), the reduction of thirst intensity after management was clinically relevant. Adverse events occurred rarely and did not require specific interventions (Table 5).

**Table 3:** Assessment of safety criteria at different thirst management time points.

**Items**	0 min		15 min		30 min		45 min	
**Failed safety criteria**	158	(17.56%)	45	(5.00%)	27	(3.00%)	7	(0.78%)
**Passed safety criteria**	742	(82.44%)	855	(95.00%)	873	(97.00%)	893	(99.22%)
***Room temperature water (n=374)***								
Eligible	319	(85.29%)	351	(93.85%)	363	(97.06%)	372	(99.47%)
Managed	262	(70.05%)	265	(70.86%)	250	(66.84%)	162	(43.32%)
***Ice cubes (n=324)***								
Eligible	265	(81.79%)	309	(95.37%)	315	(97.22%)	320	(98.77%)
Managed	187	(57.72%)	186	(57.41%)	154	(47.53%)	93	(28.70%)
***Oral Moisturizer (n=202)***								
Eligible	158	(78.22%)	195	(96.53%)	195	(96.53%)	201	(99.50%)
Managed	114	(56.44%)	138	(68.32%)	143	(70.79%)	108	(53.47%)

Safety criteria were based on an assessment of consciousness, airway reflex, and presence of nausea feeling. Data are given as number of patients (percentages in parentheses).

**Table 4:** Generalized estimating equation model for post-operative thirst management.

Parameter	Estimate	95% Wald CI		P value
		Lower bound	Upper bound	
**Management strategy**				
Room temperature water	Reference			
Ice cubes	− 0.47	− 0.97	0.04	0.072
Oral moisturizers	− 1.11	− 1.75	− 0.47	0.001
**Management strategy × time**				
Room temperature water	− 0.92	− 1.01	− 0.83	< 0.001
Ice cubes	− 0.93	− 1.06	− 0.81	< 0.001
Oral moisturizers	− 0.60	− 0.74	− 0.47	< 0.001

CI, confidence interval. The generalized estimating equation model was adjusted for age, sex, surgery type, anesthesia type, nothing-by-mouth duration, American Society of Anesthesiologists classification, operating time, body temperature, perioperative fluid balance, and medication use.

**Table 5:** Mean intensity of post-operative thirst at each time point, satisfaction scores, and number of adverse events.

	Room temperature water (n = 374)	Ice cubes (n = 324)	Oral moisturizers (n = 202)	P value
**Intensity of thirst (scale from 0 to 10)**				
T0 (at 0 min)	5.24 ± 2.50	4.83 ± 2.72	4.69 ± 3.08	
T1 (at 15 min)	4.25 ± 2.06	3.86 ± 2.38	4.21 ± 2.44	
T2 (at 30 min)	3.55 ± 1.95	3.04 ± 2.15	3.86 ± 2.11	
T3 (at 45 min)	2.51 ± 1.75	2.08 ± 1.73	2.89 ± 1.76	
**Satisfaction (scale from 1 to 5)**				
Score (at 60 min)	4.08 ± 0.55	4.22 ± 0.58	3.90 ± 0.55	0.001
**Adverse event, n (%)**				
Severe cough	4 (1.07%)	0 (0.00%)	0 (0.00%)	0.083
Vomiting	2 (0.53%)	1 (0.31%)	1 (0.50%)	1.000
Nausea	3 (0.80%)	1 (0.31%)	0 (0.00%)	0.552

The intensity of post-operative thirst and satisfaction with the intervention are expressed as means ± standard deviations. Adverse events are given as counts and percentages. Continuous variables were compared with ANOVA, whereas categorical data are compared with the Fisher’s exact test.

## Discussion

The main results of our study can be summarized as follows: (1) moderate-to-severe post-operative thirst is common (55.8%) in a high-volume PACU, (2) the use of glycopyrrolate is the main independent risk factor for moderate-to-severe post-operative thirst and (3) the use of ice cubes or distilled room temperature water is superior to an oral moisturizer for the management of post-operative thirst in a high-volume PACU.

Our data on the high prevalence of moderate-to-severe post-operative thirst in the PACU are broadly in line with the available literature and are in accordance with those reported by a large-scale cross-sectional survey conducted in UK National Health Service hospitals^2^. Despite its high frequency, there is still no clear consensus on the optimal management of post-operative thirst. The results of our GEE model demonstrated that both ice cubes and room temperature water outperformed an oral moisturizer in terms of efficacy, without significant differences between the first two options. These findings confirm previous data on the key role of cold temperature in mediating pre-absorptive thirst satiation^4,5^. From a practical standpoint, the use of room temperature water may be preferable over ice cubes—whose preparation would pose a higher workload on the PACU nurses. Moreover, room temperature water is more easily accessible and does not require the use of refrigerators. Based on its limited efficacy, the use of oral moisturizers (wet swabs or water gargling) for post-operative thirst should be limited to (1) patients with strict nothing-by-mouth order, (2) cases who need to remain in the supine position, or (3) patients who specifically make a request for an oral moisturizer. Although no consensus criteria exist for minimally important clinical difference (MCID) in the assessment of thirst intensity, an NRS reduction of 1.7 points has been used as MCID in a previous study focusing on thirst management^8^. All of the three approaches implemented in our study reached the MCID.

An important finding of our study is the independent association between the use of the anticholinergic drug glycopyrrolate during anesthesia and the occurrence of moderate-to-severe post-operative thirst. Anticholinergic drugs are increasingly being used in the premedication phase before small surgery^19^ because of their capacity to reduce secretions, protect against vagal stimulation^20^, facilitate tracheal intubation, and provide a better visualization^21^. Glycopyrrolate is generally preferred to atropine because of its ability to inhibit saliva production and its more favorable safety profile (being characterized by a lower risk of cardiovascular or central nervous system adverse events)^22,23^. Moreover, premedication with glycopyrrolate may reduce sore throat after general endotracheal anesthesia, post-operative catheter-related bladder discomfort following general anesthesia, and the frequency and intensity of nausea, as well as the severity of hypotensive episodes during spinal anesthesia^24–28^. However, one study reported that only 2% of patients who did not undergo premedication experienced excess secretion that required treatment^29^. Although the benefits and optimal dose of glycopyrrolate has been the subject of continuing debate^30–32^, this drug should be used judiciously because of its prolonged antisialagogue effect.

The perception of thirst involves a number of components—including dry mouth, dry lips, thick tongue, thick saliva, dry throat, bad taste, and desire to drink water—which may have subtle distinctions^33^. The use of different scales to assess perioperative thirst discomfort may lead to variations in its prevalence across different studies. For example, the frequency of dry mouth in the post-operative period may be as high as 85%^34,35^. However, a correct assessment of the various facets of thirst may be difficult in the early post-operative period^36^. We therefore resorted to a simple assessment tool based on an NRS. Most of the patients who declined thirst management had low thirst scores (Table 6), which may explain their unwillingness to receive thirst alleviation strategies.

**Table 6:** Mean intensity of post-operative thirst at each time point in patients who did not receive thirst management.

	No thirst feeling at PACU (n = 122)	Refusal despite mild thirst feeling (n = 153)	Refusal despite moderate-to-severe thirst feeling (n = 34)	Failure of safety protocol (n = 2)
**Severity of thirst (scale from 0 to 10)**				
T0 (at 0 min)	0.00	1.67 ± 1.06	3.61 ± 2.06	5.00 ± 0.00
T1 (at 15 min)	0.00	1.66 ± 1.00	3.58 ± 2.08	3.50 ± 3.54
T2 (at 30 min)	0.00	1.65 ± 0.96	3.79 ± 1.69	3.50 ± 3.54
T3 (at 45 min)	0.00	1.61 ± 0.95	3.85 ± 1.67	3.50 ± 3.54

The intensity of post-operative thirst is expressed as means ± standard deviations.

A previous prospective randomized trial demonstrated that early oral hydration in the PACU (with volume restricted to 0.5 mL/kg) is well-tolerated in the post-operative period (time to the first drink: 0.29 ± 0.14 h)^9^, with reported rates of nausea and vomiting of 7.6% and 4.5%, respectively. Our study confirms that early hydration with small and separated volumes of ice cubes or room temperature water is safe for the management of post-operative thirst in a high-volume PACU.

Several limitations of our study merit comment. First, the retrospective nature of our research allows identifying associations—not prediction or causation. Second, allocation to a specific thirst management strategy was not randomized. Third, our study did not include a control group. Our overarching goal was to optimize thirst management in the majority of patients; therefore, only a small number of patients with moderate-to-severe thirst did not receive treatment. Future research should examine whether administration of higher water quantity or an increased number of ice cubes will further decrease thirst without increasing post-operative nausea and vomiting. Finally, the dose of glycopyrrolate and the reasons motivating its use during anesthesia (prophylactic use *versus* treatment of excess secretions) were not specifically recorded. Although glycopyrrolate dosage is likely to affect thirst intensity, we cannot draw conclusions on this possibility and our data need to be considered as hypothesis-generating. Further research is needed to survey the presence and intensity of post-operative thirst in patients receiving glycopyrrolate administration during surgery.

## Conclusion

Moderate-to-severe post-operative thirst is commonly observed in the PACU and that the use of glycopyrrolate is the main independent risk factor. The use of ice cubes or room temperature water is superior to an oral moisturizer for the management of post-operative thirst. From a practical standpoint, the supply of room temperature water every 15 min is a simple and safe strategy that may easily implemented in the early post-operative period in a high-volume PACU.

## Data Availability

The dataset used in the current study is available from the corresponding author upon reasonable request.
